# Pump-Driven Opto-Magnetic Properties in Semiconducting Transition-Metal Dichalcogenides: An Analytical Model

**DOI:** 10.3390/nano14080707

**Published:** 2024-04-18

**Authors:** Habib Rostami, Federico Cilento, Emmanuele Cappelluti

**Affiliations:** 1 Department of Physics, University of Bath, Claverton Down, Bath BA2 7AY, UK; hr745@bath.ac.uk; 2 Elettra-Sincrotrone Trieste S.C.p.A., 34149 Basovizza, Italy; federico.cilento@elettra.eu; 3 Istituto di Struttura della Materia, CNR (ISM-CNR), 34149 Trieste, Italy

**Keywords:** transition-metal dichalcogenides, magneto-optical properties, time-resolved nonequilibrium spectroscopy, quantum entanglement, Faraday/Kerr rotation

## Abstract

Single-layer transition-metal dichalcogenides provide an unique intrinsic entanglement between the spin/valley/orbital degrees of freedom and the polarization of scattered photons. This scenario gives rise to the well-assessed optical dichroism observed by using both steady and time-resolved probes. In this paper, we provide compact analytical modeling of the onset of a finite Faraday/Kerr optical rotation upon shining with circularly polarized light. We identify different optical features displaying optical rotation at different characteristic energies, and we describe in an analytical framework the time-dependence of their intensities as a consequence of the main spin-conserving and spin-flip processes.

## 1. Introduction

The family of semiconducting transition-metal dichalcogenides (TMDs) $MX_{2}$ (M= Mo, W; X= S, Se) appears as one of the most promising platforms for future technological applications [1,2,3,4]. These materials are indeed characterized by the presence of many degrees of freedom (charge, spin, valley, layer, lattice, …), strongly entangling each other [5,6,7,8,9,10,11], opening the possibility of tuning the electronic/optical/magnetic/transport properties in a controlled, flexible, and reversible way by external magnetic or electric fields. When isolated at the single-layer level, these compounds present a direct bandgap at the high-symmetry points K, K′ of the Brillouin zone, the *valleys*, as shown by photoluminescence probes [5,7,12,13,14,15]. As in graphene, the honeycomb lattice structure is reflected in peculiar optical selection rules, which induce selectively interband optical transitions in a given valley upon circularly polarized light. This scenario prompts the concept of “valleytronics”, i.e., the possibility of manipulating the quantum degrees of freedom selectively in a single valley [13,14]. Such optical sensitivity in TMDs has been widely explored in single-layer compounds [2,4,8,16,17,18,19,20,21,22,23,24,25,26,27,28,29,30]. A common tool is the observation of an optical dichroism, i.e., a different optical response upon left-hand or right-hand circularly polarized photons. One striking difference of these compounds with respect to graphene is the presence of a strong spin–orbit coupling, which provides a sizable spin-splitting of the valence band. Within this context, circularly polarized light is selectively coupled not only with a given valley, but also with a given spin, yielding spin-polarized charges in the conduction band along with opposite-spin charges in the valence band [4,8,16,17,18,19,20,21,22,23,26,27,29,31,32,33,34,35,36].

The entanglement between light polarization and spin population can be conveniently investigated by means of the observation of a finite Kerr or Faraday rotation [37,38,39]. These effects signalize the presence of an intrinsic magnetic field in the sample, and in single-layer TMDs they can be naturally triggered as a result of circularly polarized pumping [40], which gives rise, as discussed above, to valley-selective and spin-selective particle–hole excitations [4,19,20,21,22,23,29,35,36,41,42].

The aim of the present paper is to provide a compact and microscopical investigation of the onset of Kerr/Faraday rotation in a wide energy spectrum of single-layer TMDs. A key point is the identification of the orbital character of the particle–hole excitations at different energies allowed by the optical selection rules, and how it is reflected in the sign and strength of the optical Kerr/Faraday rotation. In order to address this issue in the clearest and controlled way, we introduce a proper generalization of a k·p expansion in a three-band framework. The optical response is computed at the non-interacting level within a fully quantum Kubo approach, where Kerr/Faraday effects are related to the appearing of off-diagonal components of the optical tensor. Different optical features are identified as associated with different particle–hole excitations, and their time evolution in out-of-equilibrium dynamics is discussed. While the exact energies of such optical features should be considered as unrenormalized by exciton binding effects (not considered here), the present work provides an analytical insight into the microscopic onset of the different optical features whose strengths can be conveniently modeled in terms of a unique parameter.

## 2. Single-Particle Hamiltonian

Single-layer TMDs contain a plane of *M*-atoms in a triangular lattice, sandwiched between two layers of chalcogen atoms, *X*. The resulting lattice, from a top view, appears as a bipartite hexagonal structure. Many theoretical approaches have been proposed to capture the relevant physics of these materials. As a general rule, effective low-energy models (like k·p expansions) retain only the relevant conduction and valence bands, mapping the complex band structure onto an effective two-band gapped Dirac model [7,43,44,45]. On the other hand, tight-binding (TB) models have emphasized the role of the *d*-orbitals of the metal atoms, in particular the $d_{3z^{2}-r^{2}}$, $d_{xy}$, and $d_{x^{2}-y^{2}}$ ones, which provide the main orbital content of both the valence and conduction bands, as well as of a third higher-energy conduction band [46,47,48,49,50,51,52,53]. From a microscopical point of view, however, the simple triangular lattice of the *M* atoms cannot account for a gapped semiconducting band-structure, and the hybridization with *X* atoms has been shown to play a crucial role. The choice between a simplified, semi-analytical approach and the multiband complexity of a fully microscopical TB model is a delicate issue that depends on the physics on which to be focused.

An interesting balance between these two approaches has been provided by Liu et al. in Ref. [54], where they introduced a compact three-band tight-binding model within the reduced Hilbert space:

(1) $$\begin{array}{ccc} \phi_{p}^{†} & = & (d_{p,3z^{2}-r^{2}}^{†},d_{p,xy}^{†},d_{p,x^{2}-y^{2}}^{†},), \end{array}$$

where the role of the hybridization of the *d*-orbitals of *M* atoms with *p*-orbitals of *X* is modeled, using symmetry arguments, by means of effective complex hopping parameters that break the triangular symmetry, enforcing the physics of a bipartite hexagonal lattice. The resulting one-particle Hamiltonian can be thus written in the full Brillouin zone thus as:

(2) $${\hat{H}}_{\sigma }\left(k\right)={\hat{H}}_{\mathrm{TB}}\left(k\right)+{\hat{H}}_{\mathrm{SO},\sigma },$$

where interatomic hoppings up to third-nearest-neighbor level are included in the TB part, ${\hat{H}}_{\mathrm{TB},k}$, providing an excellent agreement with ab initio calculations for the conduction and valence bands close to the K, K′ valleys. The spin–orbit coupling (SOC) is safely approximated with its dominant contribution due to the local spin-diagonal term [54,55], which in this basis reads:

(3) $${\hat{H}}_{\mathrm{SO},\sigma }=\lambda I_{\sigma }\left(\begin{array}{ccc} 0 & 0 & 0\\ 0 & 0 & i\\ 0 & -i & 0 \end{array}\right),$$

where $I_{\sigma }=\delta_{\sigma ,↑}-\delta_{\sigma ,↓}$.

Such a three-band tight-binding model provides an accurate description of the energy dispersion and of the orbital character of the main relevant bands for optical probes with the advantage of a reduced Hilbert space. In the following, we focus on the optical response that is governed by the particle–hole excitations close to the K, K′ valley points. The above model thus also represents a suitable platform for an analytical k·p expansion that generalizes the previous k·p approaches [7,43,44,45] up to the relevant three-orbital space. To this aim, we thus expand $\hat{H}\left(k\right)$ up to the quadratic order in the relative momentum p=k−K ($p=k-K^{\prime }$) close to the K, K′. It is also convenient to express the resulting Hamiltonian $\hat{H}\left(p,K\right)$, $\hat{H}\left(p,K^{\prime }\right)$ in the chiral basis:

(4) $$\begin{array}{ccc} \psi_{p}^{†} & = & (d_{p,3z^{2}-r^{2}}^{†},d_{p,L}^{†},d_{p,R}^{†},), \end{array}$$

where $d_{p,L}=\left(d_{p,x^{2}-y^{2}}-id_{p,xy}\right)/\sqrt{2}$ and $d_{p,R}=\left(d_{p,x^{2}-y^{2}}+id_{p,xy}\right)/\sqrt{2}$. On such a basis, the spin–orbit term appears purely diagonal,

(5) $${\hat{H}}_{\mathrm{SO},\sigma }=-\lambda I_{\sigma }\left(\begin{array}{ccc} 0 & 0 & 0\\ 0 & 1 & 0\\ 0 & 0 & -1 \end{array}\right),$$

and we can write:

(6) $$\begin{array}{ccc} \hat{H}\left(p,K\right) & = & \left(\begin{array}{ccc} E_{0}+a_{0}p^{2}a^{2} & v_{0/T}\left(p_{+}a\right) & v_{0/B}\left(p_{-}a\right)\\ v_{0/T}{\left(p_{+}a\right)}^{*} & E_{T}+a_{T}p^{2}a^{2} & v_{T/B}\left(p_{+}a\right)\\ v_{0/B}{\left(p_{-}a\right)}^{*} & v_{T/B}{\left(p_{+}a\right)}^{*} & E_{B}+a_{B}p^{2}a^{2} \end{array}\right), \end{array}$$

(7) $$\hat{H}\left(p,K^{\prime }\right)=\left(\begin{array}{ccc} E_{0}+a_{0}p^{2}a^{2} & -v_{0/B}\left(p_{+}a\right) & -v_{0/T}\left(p_{-}a\right)\\ -v_{0/B}{\left(p_{+}a\right)}^{*} & E_{B}+a_{B}p^{2}a^{2} & -v_{T/B}\left(p_{+}a\right)\\ -v_{0/T}{\left(p_{-}a\right)}^{*} & -v_{T/B}{\left(p_{+}a\right)}^{*} & E_{T}+a_{T}p^{2}a^{2} \end{array}\right),$$

where $p_{\pm }=p_{x}\pm ip_{y}$ and *a* is the in-plane *M*-*M* distance. The full expression of the band parameters in Equations (6) and (7) in terms of the original tight-binding parameters is provided in Appendix A. The total spin-full Hamiltonians at the K and K′

(8) $${\hat{H}}_{\sigma }\left(p,\nu \right)=\hat{H}\left(p,\nu \right)+{\hat{H}}_{\mathrm{SO},\sigma },$$

(where ν= K, K′) contain all the relevant entanglements between spin, valleys, and chirality.

Equation (8) defines the energy levels at the K point for each spin family. We have explicitly:

(9) $$\begin{array}{ccc} \epsilon_{T,\sigma }\left(0\right) & = & E_{T}-\lambda I_{\sigma }, \end{array}$$

(10) $$\begin{array}{ccc} \epsilon_{0,\sigma }\left(0\right) & = & E_{0}, \end{array}$$

(11) $$\begin{array}{ccc} \epsilon_{B,\sigma }\left(0\right) & = & E_{B}+\lambda I_{\sigma }. \end{array}$$

At the K point, the energy level $E_{0}$ corresponds to the conduction band edge, which results here in spin-degeneracy since we neglect the weak spin–orbit coupling of the *X* chalcogen atoms. The valence band at K is associated with the R-chiral state with spin-split energies $E_{B}\pm \lambda$, for up and down spin, respectively. The $E_{T}\pm \lambda$ levels correspond to higher energy states, characterized by a L-chiral symmetry [47]. A similar energy spectrum is found at the K′ point, but with chiral content exchanged between the valence and the high-energy levels.

The optical selection rules are encoded in the multiband matrix structure of the current operators, which can be straightforwardly computed as derivatives of the single-particle Hamiltonian, ${\hat{J}}_{i}\left(p,\nu \right)=dH\left(p,\nu \right)/dp_{i}$, where i=x,y and ν= K, K′. At the high-symmetry points (p=0) we obtain:

(12) $$\begin{array}{ccc} {\hat{J}}_{x}\left(K\right) & = & \left(\begin{array}{ccc} 0 & v_{0/T}a & v_{0/B}a\\ v_{0/T}a & 0 & v_{T/B}a\\ v_{0/B}a & v_{T/B}a & 0 \end{array}\right), \end{array}$$

(13) $$\begin{array}{ccc} {\hat{J}}_{y}\left(K\right) & = & \left(\begin{array}{ccc} 0 & iv_{0/T}a & -iv_{0/B}a\\ -iv_{0/T}a & 0 & iv_{T/B}a\\ iv_{0/B}a & -iv_{T/B}a & 0 \end{array}\right), \end{array}$$

(14) $$\begin{array}{ccc} {\hat{J}}_{x}\left(K^{\prime }\right) & = & -\left(\begin{array}{ccc} 0 & v_{0/B}a & v_{0/T}a\\ v_{0/B}a & 0 & v_{T/B}a\\ v_{0/T}a & v_{T/B}a & 0 \end{array}\right), \end{array}$$

(15) $$\begin{array}{ccc} {\hat{J}}_{y}\left(K^{\prime }\right) & = & \left(\begin{array}{ccc} 0 & -iv_{0/B}a & iv_{0/T}a\\ iv_{0/B}a & 0 & -iv_{T/B}a\\ -iv_{0/T}a & iv_{T/B}a & 0 \end{array}\right). \end{array}$$

In similar way, one can derive the chiral current operators ${\hat{J}}_{\pm }\left(\nu \right)={\hat{J}}_{x}\left(\nu \right)\pm i{\hat{J}}_{x}\left(\nu \right)$:

(16) $$\begin{array}{ccc} {\hat{J}}_{+}\left(K\right) & = & \left(\begin{array}{ccc} 0 & 0 & 2v_{0/B}a\\ 2v_{0/T}a & 0 & 0\\ 0 & 2v_{T/B} & 0 \end{array}\right), \end{array}$$

(17) $$\begin{array}{ccc} {\hat{J}}_{-}\left(K\right) & = & \left(\begin{array}{ccc} 0 & 2v_{0/T}a & 0\\ 0 & 0 & 2v_{T/B}\\ v_{0/B}a & 0 & 0 \end{array}\right), \end{array}$$

(18) $$\begin{array}{ccc} {\hat{J}}_{+}\left(K^{\prime }\right) & = & \left(\begin{array}{ccc} 0 & 0 & -2v_{0/T}a\\ -v_{0/B}a & 0 & 0\\ 0 & -v_{T/B}a & 0 \end{array}\right), \end{array}$$

(19) $$\begin{array}{ccc} {\hat{J}}_{-}\left(K^{\prime }\right) & = & \left(\begin{array}{ccc} 0 & -2v_{0/B}a & 0\\ 0a & 0 & -2v_{T/B}a\\ -2v_{0/T}a & 0a & 0 \end{array}\right). \end{array}$$

In order to evaluate the optical response, Equation (8) can be numerically diagonalized for finite momentum p to obtain eigenvalues (the band dispersion) and eigenvectors of the resulting eigenstates. This task, however, can be further simplified within a k·p framework where the band dispersion, at the quadratic order we are interested in, is simply provided by the diagonal terms of Equations (6) and (7) *corrected* by the second-order corrections resulting from the off-diagonal elements. We thus obtain:

(20) $$\begin{array}{ccc} {\hat{H}}_{\sigma }\left(p,\nu \right) & \approx & {\hat{E}}_{\sigma }\left(p,\nu \right), \end{array}$$

where

(21) $$\begin{array}{ccc} {\hat{E}}_{\sigma }\left(p,K\right) & = & \left(\begin{array}{ccc} \epsilon_{0,\sigma }\left(p\right) & 0 & 0\\ 0 & \epsilon_{T,\sigma }\left(p\right) & 0\\ 0 & 0 & \epsilon_{B,\sigma }\left(p\right) \end{array}\right), \end{array}$$

(22) $$\begin{array}{ccc} {\hat{E}}_{\sigma }\left(p,K^{\prime }\right) & = & \left(\begin{array}{ccc} \epsilon_{0,-\sigma }\left(p\right) & 0 & 0\\ 0 & \epsilon_{B,-\sigma } & 0\\ 0 & 0 & \epsilon_{T,-\sigma }\left(p\right) \end{array}\right), \end{array}$$

and where

(23) $$\begin{array}{ccc} \epsilon_{T,\sigma }\left(p\right) & = & E_{T}-\lambda I_{\sigma }+{\bar{a}}_{T,\sigma }p^{2}a^{2}, \end{array}$$

(24) $$\begin{array}{ccc} \epsilon_{0,\sigma }\left(p\right) & = & E_{0}+{\bar{a}}_{0,\sigma }p^{2}a^{2}, \end{array}$$

(25) $$\begin{array}{ccc} \epsilon_{B,\sigma }\left(p\right) & = & E_{B}+\lambda I_{\sigma }+{\bar{a}}_{B,\sigma }p^{2}a^{2}. \end{array}$$

The numerical expression of the k·p band parameters in Equations (23)–(25) is also provided in Appendix A.

**Figure 1:**
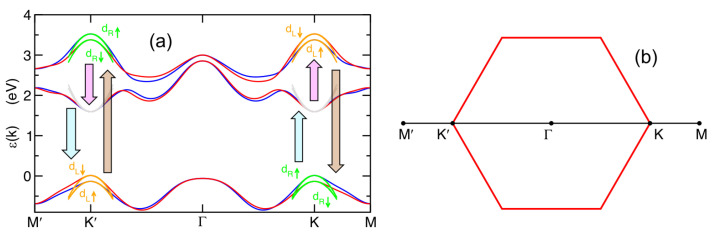
(**a**) Comparison between the three-band TB model from Ref. [54] and our analytical three-band k·p model for single-layer MoS_2_ along the path described in panel (**b**). Blue and red solid lines represent the TB band-dispersion for spin-up and spin-down electrons, respectively. Green, orange, and gray lines show the band-dispersions for the eigenstates with orbital character $d_{R}$, $d_{L}$, and $d_{3z^{2}-r^{2}}$, respectively. The vertical arrows represent the optical interband transitions allowed at the K, K′ points by a left-circularly polarized photon.

The comparison between the full TB dispersion in the Brillouin zone, from Ref. [54], and our analytical three-band model expanded around the K, K′ points is displayed in Figure 1, showing an excellent agreement.

Note that, within the same k·p expansion, the leading order to the current operators is not affected and Equations (16)–(19) are still valid also in the k·p context. As mentioned, the different matrix structure of Equations (16)–(19) enforces the different optical selection rules at the K and K′ points. It is useful to recall that right-circularly polarized (RCP) light couples with the L-chiral current $J_{-}$, whereas LCP couples with $J_{+}$, according to the relation $A_{x}J_{x}+A_{y}J_{y}=\left[A_{+}J_{-}+A_{-}J_{+}\right]/2$. Equations (16)–(19) dictate for instance how, under external pumping conditions, absorption of a left-circularly polarized (LCP) photon can induce particle–hole optical excitations at the K point only between the valence band with $d_{R}$ character and the conduction band with $d_{3z^{2}-r^{2}}$ character, or (in the case of electron-doped samples) between the conduction band with $d_{3z^{2}-r^{2}}$ character and the high-level conduction band with $d_{L}$ character. On the other hand, the same absorption of a LCP photon can effectively create particle–hole optical excitations at the K′ point between the valence band with $d_{R}$ character and the high-level conduction band with $d_{L}$ character. The selection rules for right-circularly polarized light, coupled with the chiral current $J_{+}$, are graphically obtained by reversing the arrow of each particle–hole excitation.

Similar selection rules govern also the virtual processes involved in the optical linear response, whose analytical computation will be addressed in the next section.

## 3. Optical Response

Equipped with the theoretical tools outlined in Section 2, we can now compute in the useful chiral basis all the elements of the optical tensor of single-layer transition-metal dichalcogenides through the evaluation of the frequency-dependent current–current response function. In the Matsubara space we have:

(26) $$\begin{array}{ccc} \pi_{ij}\left(i\hbar \omega_{m},\nu \right) & = & \frac{e^{2}T}{4\pi^{2}\hbar^{2}}\sum_{\sigma ,n}\int d^{2}pTr\left[{\hat{J}}_{i}\left(\nu \right){\hat{G}}_{\sigma }\left(p,i\omega_{n}+i\omega_{m},\nu \right){\hat{J}}_{j}^{†}\left(K\right){\hat{G}}_{\sigma }\left(p,i\omega_{n},\nu \right)\right], \end{array}$$

where i,j=x,y, ν= K, K′,

(27) $$\begin{array}{ccc} {\hat{G}}_{\sigma }\left(p,z,\nu \right) & = & \frac{1}{\left(z+\mu \right)\hat{I}-{\hat{E}}_{\sigma }\left(p,\nu \right)}, \end{array}$$

and where μ is the chemical potential. Here, $\hbar \omega_{n}=\pi T\left(2n+1\right)$ are the internal fermionic frequencies, $\hbar \omega_{m}=2\pi Tm$ is the external bosonic frequency, and *T* is the temperature.

The generic elements of the optical conductivity tensor are thus obtained as:

(28) $$\begin{array}{ccc} \sigma_{ij}\left(\omega ,\nu \right) & = & -\frac{\pi_{ij}\left(\hbar \omega ,\nu \right)}{i\omega }, \end{array}$$

where

(29) $$\begin{array}{ccc} \pi_{ij}\left(\hbar \omega ,\nu \right) & = & \pi_{ij}\left(i\hbar \omega_{m},\nu \right){|}_{i\omega_{m}\to \omega }. \end{array}$$

Given the three-band structure of our model, the total optical response can be divided (leaving aside the Drude-like intraband terms at low frequencies, which are not relevant in the present analysis) in three interband contributions:

(30) $$\begin{array}{ccc} \sigma_{ij}\left(\omega ,\nu \right) & = & \sigma_{ij}^{0,T}\left(\omega ,\nu \right)+\sigma_{ij}^{0,B}\left(\omega ,\nu \right)+\sigma_{ij}^{T,B}\left(\omega ,\nu \right). \end{array}$$

The term $\sigma_{ij}^{0,B}$ describes optical transitions between the (spin-split) valence band and the lowest energy conduction bands. Due to the spin-splitting of the valence band, this term accounts for the A and B exciton resonances. The term $\sigma_{ij}^{T,B}$ conveys information about optical transitions between the valence band (with $d_{R}$ or $d_{L}$ character) and the high-energy conduction band with opposite $d_{R}/d_{L}$ character, which has been discussed in detail in Refs. [26,27]. Finally, the term $\sigma_{ij}^{0,T}$ describes optical transitions between the lowest-energy and high-energy conductions bands. In the undoped semiconducting regime, due to the Pauli blocking, this term is usually irrelevant, but it plays a role in photo-induced doping [26,27].

All the terms present a similar functional structure, where the relevant role is played by the band population. For sake of simplicity, we focus thus for the moment on the first term, $\sigma_{ij}^{0,B}$. Since the system is block-diagonal in the spin-index, one can also formally compute separately the optical response $\sigma_{ij,\sigma }\left(\omega ,\nu \right)$ for each spin and each valley, ν. We have for instance:

(31) $$\begin{array}{ccc} \sigma_{xx,\sigma }^{0,B}\left(\omega ,K\right) & \approx & -\frac{e^{2}}{4\pi^{2}\hbar^{2}}\frac{{\left(v_{0/B}a\right)}^{2}}{\omega }\int d^{2}p[\frac{f\left[\epsilon_{0,\sigma }\left(p\right)\right]-f\left[\epsilon_{B,\sigma }\left(p\right)\right]}{\epsilon_{0,\sigma }\left(p\right)-\epsilon_{B,\sigma }\left(p\right)-\omega -i\delta }\\ & & +\frac{f\left[\epsilon_{0,\sigma }\left(p\right)\right]-f\left[\epsilon_{B,\sigma }\left(p\right)\right]}{\epsilon_{0,\sigma }\left(p\right)-\epsilon_{B,\sigma }\left(p\right)+\omega +i\delta }], \end{array}$$

where f[E] is the occupation factor for a given momentum and band, where, under equilibrium conditions, f[E]=1/{exp[(E−μ)/T]+1}. At T=0, in the semiconducting state μ=0, one obtains $f[\epsilon_{0,\sigma }\left(p\right)]=0$, $f[\epsilon_{B,\sigma }\left(p\right)]=1$, and, for ω>0:

(32) $$\begin{array}{ccc} Re\sigma_{xx,↑}^{0,B}\left(\omega ,K\right) & = & \sigma_{0}\frac{v_{0/B}^{2}}{c_{A}\hbar \omega }\theta \left(\hbar \omega -\Delta_{A}\right), \end{array}$$

(33) $$\begin{array}{ccc} Re\sigma_{xx,↓}^{0,B}\left(\omega ,K\right) & = & \sigma_{0}\frac{v_{0/B}^{2}}{c_{B}\hbar \omega }\theta \left(\hbar \omega -\Delta_{B}\right), \end{array}$$

where $\sigma_{0}=e^{2}/4\hbar$ is the universal two-dimensional conductivity, $\Delta_{A}$, $\Delta_{B}$ and the excitation edges for the A and B excitons, respectively,

(34) $$\begin{array}{ccc} \Delta_{A} & = & E_{0}-E_{B}-\lambda , \end{array}$$

(35) $$\begin{array}{ccc} \Delta_{B} & = & E_{0}-E_{B}+\lambda , \end{array}$$

and

(36) $$\begin{array}{ccc} c_{A} & = & {\bar{a}}_{0,↑}-{\bar{a}}_{B,↑}, \end{array}$$

(37) $$\begin{array}{ccc} c_{B} & = & {\bar{a}}_{0,↓}-{\bar{a}}_{B,↓}. \end{array}$$

Using the tight-binding parameters of Ref. [54], we obtain for MoS_2_ $\Delta_{A}=1.584$ eV, $\Delta_{B}=1.730$ eV, $v_{0/B}=-1.545$, $c_{A}=1.598$ eV, and $c_{B}=1.616$ eV. For symmetry, we have also $\sigma_{xx,\sigma }\left(\omega ,\nu \right)=\sigma_{yy,\sigma }\left(\omega ,\nu \right)$.

In a similar way, one obtains:

(38) $$\begin{array}{ccc} \sigma_{xy,\sigma }^{0,B}\left(\omega ,K\right) & = & i\frac{e^{2}}{4\pi^{2}\hbar^{2}}\frac{{\left(v_{0/B}a\right)}^{2}}{\omega }\sum_{p}[\frac{f\left[\epsilon_{0,\sigma }\left(p\right)\right]-f\left[\epsilon_{B,\sigma }\left(p\right)\right]}{\epsilon_{0,\sigma }\left(p\right)-\epsilon_{B,\sigma }\left(p\right)-\omega -i\delta }\\ & & -\frac{f\left[\epsilon_{0,\sigma }\left(p\right)\right]-f\left[\epsilon_{B,\sigma }\left(p\right)\right]}{\epsilon_{0,\sigma }\left(p\right)-\epsilon_{B,\sigma }\left(p\right)+\omega +i\delta }], \end{array}$$

and, for T,μ=0, and ω>0:

(39) $$\begin{array}{ccc} Im\sigma_{xy,↑}^{0,B}\left(\omega ,K\right) & = & -\sigma_{0}\frac{v_{0/B}^{2}}{c_{A}\hbar \omega }\theta \left(\hbar \omega -\Delta_{A}\right), \end{array}$$

(40) $$\begin{array}{ccc} Im\sigma_{xy,↓}^{0,B}\left(\omega ,K\right) & = & -\sigma_{0}\frac{v_{0/B}^{2}}{c_{B}\hbar \omega }\theta \left(\hbar \omega -\Delta_{B}\right). \end{array}$$

**Figure 2:**
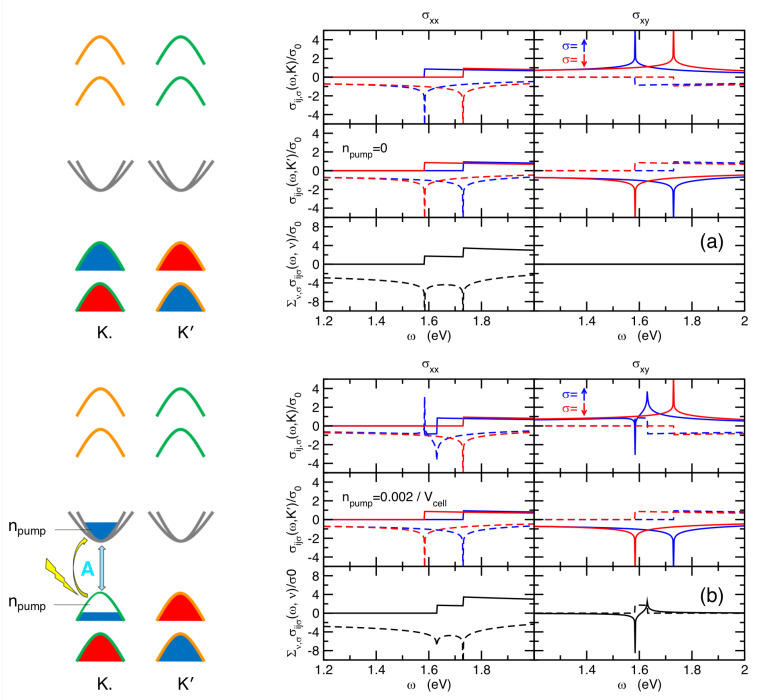
Plot of the different contributions to the diagonal, $\sigma_{xx}$, and off-diagonal, $\sigma_{xy}$, parts of the optical conductivity of single-layer TMDs (e.g., MoS_2_ here) resolved for different valleys and different spins. Panel (**a**) represents the semiconducting equilibrium case and panel (**b**) the non-equilibrium case induced by a left-circularly polarized pumping tuned at the $\omega =\Delta_{A}$ frequency, which acts only on the top valence band at the K point. Blue and red lines represent contributions from spin-up and spin-down, respectively, whereas black lines represent the total optical conductivity summed up over spin and valleys.

The real parts of $\sigma_{xy,\sigma }\left(\omega ,K\right)$ and the imaginary parts of $\sigma_{xx,\sigma }\left(\omega ,K\right)$ can be thus easily obtained using the Kramers–Kronig relations. Furthermore, using the symmetry relations encoded in the different matrix structures at different valleys, one can recognize that at equilibrium:

(41) $$\begin{array}{ccc} \sigma_{xx,\sigma }^{0,B}\left(\omega ,K^{\prime }\right) & = & \sigma_{xx,-\sigma }^{0,B}\left(\omega ,K\right), \end{array}$$

(42) $$\begin{array}{ccc} \sigma_{xy,\sigma }^{0,B}\left(\omega ,K^{\prime }\right) & = & -\sigma_{xy,-\sigma }^{0,B}\left(\omega ,K\right), \end{array}$$

(−σ being here the reversed σ spin), so that, under such equilibrium conditions, the contributions of different valleys *sum up* for the diagonal terms of the optical tensor, whereas they *cancel out* for the off-diagonal ones, in accordance with the time-reversal symmetry. The net result for the whole optical tensor is summarized in Figure 2a. Similar expressions can be derived for the $\sigma_{xy,\sigma }^{0,T}\left(\omega ,\nu \right)$ and $\sigma_{xy,\sigma }^{T,B}\left(\omega ,\nu \right)$ terms.

## 4. Non-Equilibrium Optical Response

Equations (31) and (38) provide a suitable framework to model the optical response under non-equilibrium conditions, by specifying the proper occupation factors in the presence of photo-induced particle–hole excitations. More specifically, within such a semi-classical approach, we can simulate the effects of an LCP laser pumping tuned at the $\omega \approx \Delta_{A}$ frequency by assuming an effective photo-induced charge transfer, $n_{\mathrm{pump}}$, from the valence to the conduction band. Due to the selected circular polarization, such particle–hole excitations occur only at the K point and, due to the selected frequency in resonance with the A exciton, only for the spin ↑. Since only one valley, with a given spin, will be populated in both the valence and conduction band, the photo-induced charge density can be further parametrized in terms of a characteristic momentum, $\bar{p}$, for which $f[\epsilon_{0,\sigma }\left(p\right)]=1$, $f[\epsilon_{B,\sigma }\left(p\right)]=0$ ($p\leq \bar{p}$), obeying the relation:

(43) $$\begin{array}{ccc} n_{\mathrm{pump}} & = & \frac{{\bar{p}}^{2}}{4\pi }. \end{array}$$

Typical values of $n_{\mathrm{pump}}$ can range up to $n_{\mathrm{pump}}≲0.01/V_{\mathrm{cell}}$. For a representative case, $n_{\mathrm{pump}}=0.002/V_{\mathrm{cell}}$, we obtain for MoS_2_ $\bar{p}a\approx 0.171$, and, using a=3.16 Å, $\bar{p}\approx 0.054$ Å^−1^.

Due to the selection rules, only the states at the K point with the proper chirality, corresponding in this case to spin-up, are affected by the pumping. For $\left|p\right|\leq \bar{p}$, we have thus a *reverse* Pauli blocking. The contribution of these states to the optical conductivity reads thus:

(44) $$\begin{array}{ccc} Re\sigma_{xx,↑,\mathrm{pump}}^{0/B}\left(\omega ,K\right) & = & \sigma_{0}\frac{v_{0/B}^{2}}{c_{A}\hbar \omega }\left[-\theta \left(\hbar \omega -\Delta_{A}\right)+2\theta \left(\hbar \omega -\Delta_{A}-E_{P,A}\right)\right], \end{array}$$

(45) $$\begin{array}{ccc} Im\sigma_{xy,↑,\mathrm{pump}}^{0/B}\left(\omega ,K\right) & = & -\sigma_{0}\frac{v_{0/B}^{2}}{c_{A}\hbar \omega }\left[-\theta \left(\hbar \omega -\Delta_{A}\right)+2\theta \left(\hbar \omega -\Delta_{A}-E_{P,A}\right)\right], \end{array}$$

where $E_{P,A}=c_{A}{\left(\bar{p}a\right)}^{2}=4\pi c_{A}a^{2}n_{\mathrm{pump}}$. In Figure 2b, we show the effect of the left-circularly polarized pumping on the diagonal and off-diagonal parts of the optical conductivity, $\sigma_{ij,\mathrm{pump}}\left(\omega \right)=\sum_{\sigma ,\nu }\sigma_{xx,\sigma ,\mathrm{pump}}^{\prime }\left(\omega ,\nu \right)$. Due to the reverse Pauli blocking, the real part of the diagonal term, Re$\sigma_{xx,\mathrm{pump}}\left(\omega \right)$, shows a depletion of spectral intensity close to the A-edge energy. In real samples, in the presence of many-body exciton binding, this depletion appears as a reduction of the A-exciton intensity, as has been experimentally observed many times in reflectivity probes. More striking is the result on the off-diagonal component of the optical tensor, $\sigma_{xy,\mathrm{pump}}\left(\omega \right)$, where the contributions from spin-up and spin-down transitions and from K and K′ close to the A-resonance do not cancel out anymore, giving rise to a *finite* off-diagonal term, $\sigma_{xy,\mathrm{pump}}\left(\omega \right)\neq 0$,

(46) $$\begin{array}{ccc} Im\sigma_{xy,\mathrm{pump}}^{0/B}\left(\omega \right) & = & \sigma_{0}\frac{2v_{0/B}^{2}}{c_{A}\hbar \omega }\left[\theta \left(\hbar \omega -\Delta_{A}\right)-\theta \left(\hbar \omega -\Delta_{A}-E_{P,A}\right)\right]. \end{array}$$

On the experimental ground, the onset of a finite off-diagonal component, $\sigma_{xy,\mathrm{pump}}\left(\omega \right)\neq 0$, is observed as a optical (Faraday or Kerr) rotation of the transmitted/reflected polarization of the probe, which commonly signalizes the presence of a finite effective magnetic field [37,38,39]. In more detail, we estimate an off-diagonal spectral intensity at the A-exciton energy range:

(47) $$\begin{array}{ccc} I_{A} & = & \int_{\Omega_{A}}d\omega \;Im\sigma_{xy,\mathrm{pump}}^{0/B}\left(\omega \right)=\sigma_{0}\frac{v_{0/B}^{2}}{\hbar c_{A}\Delta_{A}}2E_{P,A}=4\pi a^{2}\sigma_{0}\frac{v_{0/B}^{2}}{\hbar \Delta_{A}}2n_{\mathrm{pump}}, \end{array}$$

where the spectral off-diagonal intensity is meant to be integrated in the frequency range $\Omega_{A}$ close to the A-resonance. We stress here that, although we employ here a non-interacting model to obtain an analytical insight, the physical origin of such magneto-optical rotation depends uniquely on the selective valley-population enforced by the circularly polarized pumping, yielding a non-equivalent optical response that does not cancel in the K and K′ valleys. This is a quite general mechanism that will not be affected by the formation of localized states when bound excitons form. Within this context, we expect that the step-function spectral shape of Equation (46), also shown in Figure 2b, will evolve smoothly in a δ-like Lorentzian peak, preserving an integrated intensity, $I_{A}$, which is dictated by the amount of the spin-polarized photo-induced charges in the conduction and valence bands, and hence still scaling with $n_{\mathrm{pump}}$. The onset of a Faraday/Kerr rotation at the A-exciton energy is consistent with previous experimental and theoretical investigations [2,19,20,21,22,23,39]. It is worth underlining here that the valley-selective/spin-selective population induced by the circularly polarized pumping is expected within our modeling to give rise to a finite pump-driven Kerr/Faraday rotation also at two further energy scales, which we identify with the so-called C-exciton and with another characteristic energy, which we denote as the D-peak.

We notice a *remarkably strong* band-nesting between these two bands close to the K/K′ points. Such a feature has usually been disregarded in the context of TMDs, where the analyses have focused on the nesting properties between the valence and the lowest-energy conduction band [56,57,58]. We relate these transitions with the broad shoulder, commonly denoted as the C-exciton.

Currently, the origin of the remarkable shoulder in the optical conductivity denoted as the C-exciton has not been fully assessed. A mainstream consensus associates this spectral feature with the enhanced optical activity between the valence band and the lowest-energy conduction band along the Γ-K path, where these two bands are thought to have a parallel energy dispersion (*band nesting*) [56,57,58]. Generalizing this idea within the three-band context, we notice a *remarkably strong* band-nesting at K/K′ points between the valence and the lowest-energy conduction, governed by the nesting factor $∼1/|\omega -\epsilon_{T,\sigma }\left(p\right)+\epsilon_{B,\sigma }\left(p\right)|$. Prompted by a careful analysis of the first-principle and tight-binding dispersions, we suggest thus a slightly different perspective, where the C-exciton shoulder stems from band-nesting close to the K (K′) point between the valence band with $d_{R}$ ($d_{L}$) character and the high-energy conduction band with $d_{L}$ ($d_{R}$) character. In our modeling, neglecting the exciton binding energy, we can expect thus $\Delta_{C}=E_{T}-E_{B}-2\lambda$. Such a change of perspective has a deep impact on the predictions about the effects of pumping with circularly polarized light on the magneto-optical properties. In the original scenario, the k points responsible for the band-nesting are located close to the Γ point along the path Γ-K. These states do not have a significant chiral character, and as a consequence they have a small spin-splitting and they are weakly entangled with circularly polarized light. On the contrary, in the present context where band-nesting states lie close to the K, K′ points, we can predict a strong chiral character, a different response for spin-up and spin-down charges, a strong entanglement with the circularly polarized light, and a remarkable onset of a off-diagonal component of the optical tensor. Such a picture is consistent with the experimental findings observed in Refs. [26,27].

**Figure 3:**
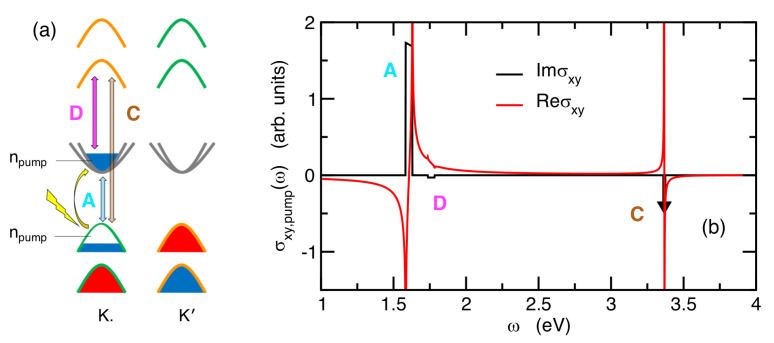
(**a**) Sketch of the optical processes responsible for the Faraday/Kerr rotation at different energies driven by the circularly polarized pumping, which is parametrized in terms of a charge density, $n_{\mathrm{pump}}$, transferred from the valence to the conduction band at the K point. The vertical light-blue arrow marks the transitions associated with the A-exciton from the valence band with orbital character $d_{R}$ to the lowest conduction band with $d_{3z^{2}-r^{2}}$ character. The brown arrow marks the transitions associated with the C-exciton from the valence band with orbital character $d_{R}$ to the high-energy conduction band with $d_{L}$ character. These processes profit from the strong band-nesting between these two bands close to the K, K′ points. The magenta arrow marks the transitions giving rise to an additional optical feature, denoted as the D-peak, corresponding to the transitions between the lowest conduction band with $d_{3z^{2}-r^{2}}$ character and the high-energy $d_{L}$-band. (**b**) Real and imaginary part of the off-diagonal component of the optical tensor, $\sigma_{xy,\mathrm{pump}}\left(\omega \right)$, as driven by the circularly polarized pumping. Due to the strong band-nesting, the C-exciton feature in Im$\sigma_{xy,\mathrm{pump}}$ is too narrow and sharp to be visible on this scale, and it has been represented by the thick black arrow.

Our three-band model is naturally suited to describe this scenario, where the band-nesting optical transitions responsible for the C-exciton shoulder are accounted for by the $\sigma_{ij,\mathrm{pump}}^{T/B}\left(\omega \right)$ term (see Figure 3a).

At the same time, the photo-induced charging of the conduction band triggers finite optical transitions between the conduction band itself with $d_{3z^{2}-r^{2}}$ and the high-energy conduction band with $d_{L}$ ($d_{R}$) character. The valley-population of these states is also very sensitive to circularly polarized light, and it is expected thus to drive a finite optical rotation at typical energy, neglecting the exciton binding energy, $\Delta_{D}=E_{T}-E_{0}-\lambda =\Delta_{C}-\Delta_{A}$ (Figure 3a). These latter optical transitions are taken into account by the term $\sigma_{ij,\mathrm{pump}}^{0/T}\left(\omega \right)$.

The effect of photo-induced pump charging with circularly polarized light in the whole frequency domain can be thus evaluated by considering all the possible contributions, $\sigma_{ij,\mathrm{pump}}\left(\omega \right)=\sigma_{ij,\mathrm{pump}}^{0/B}\left(\omega \right)+\sigma_{ij,\mathrm{pump}}^{T/B}\left(\omega \right)+\sigma_{ij,\mathrm{pump}}^{0/T}\left(\omega \right)$. The formal structure of each term is very similar to the term $\sigma_{ij,\mathrm{pump}}^{0/B}\left(\omega \right)$, which we have explicitly evaluated above. Taking into account the slight differences for each term, we obtain thus:

(48) $$\begin{array}{ccc} Im\sigma_{xy,\mathrm{pump}}\left(\omega \right) & = & \sigma_{0}\frac{2v_{0/B}^{2}}{c_{A}\hbar \omega }\left[\theta \left(\hbar \omega -\Delta_{A}\right)-\theta \left(\hbar \omega -\Delta_{A}-E_{P,A}\right)\right]\\ & & -\sigma_{0}\frac{v_{T/B}^{2}}{\left|c_{C}\right|\hbar \omega }\left[\theta \left(\hbar \omega -\Delta_{C}\right)-\theta \left(\hbar \omega -\Delta_{C}-E_{P,C}\right)\right]\\ & & -\sigma_{0}\frac{v_{0/T}^{2}}{\left|c_{D}\right|\hbar \omega }\left[\theta \left(\hbar \omega -\Delta +E_{P,D}\right)-\theta \left(\hbar \omega -\Delta_{D}\right)\right], \end{array}$$

where

(49) $$\begin{array}{ccc} c_{C} & = & {\bar{a}}_{T,↑}-{\bar{a}}_{B,↑}, \end{array}$$

(50) $$\begin{array}{ccc} c_{D} & = & {\bar{a}}_{T,↑}-{\bar{a}}_{0,↑}, \end{array}$$

and where $E_{P,C}=|c_{C}|{\left(\bar{p}a\right)}^{2}=4\pi \left|c_{C}\right|a^{2}n_{\mathrm{pump}}$ and $E_{P,D}=\left|c_{D}\right|{\left(\bar{p}a\right)}^{2}=4\pi \left|c_{D}\right|a^{2}n_{\mathrm{pump}}$. The real part, $Re\sigma_{xy,\mathrm{pump}}\left(\omega \right)$, is thus obtained by Kramers–Kronig relations. In Equation (48), we have assumed that $c_{C}>0$, which is a valid assumption for the W-based transition-metal dichalcogenides WS_2_, WSe_2_, and WTe_2_ (see Table A1). However, since these materials are very close to the perfect band-nesting limit ($c_{C}\approx 0$) for these states at the K, K′ point, the sign of $c_{C}$ is not a priori determined. For the Mo$X_{2}$ family, for instance $c_{C}<0$ (see Table A1), the analytical expression $Im\sigma_{xy,\mathrm{pump}}\left(\omega \right)$ should rather read:

(51) $$\begin{array}{ccc} Im\sigma_{xy,\mathrm{pump}}\left(\omega \right) & = & \sigma_{0}\frac{2v_{0/B}^{2}}{c_{A}\hbar \omega }\left[\theta \left(\hbar \omega -\Delta_{A}\right)-\theta \left(\hbar \omega -\Delta_{A}-E_{P,A}\right)\right]\\ & & -\sigma_{0}\frac{v_{T/B}^{2}}{\left|c_{C}\right|\hbar \omega }\left[\theta \left(\hbar \omega -\Delta_{C}+E_{P,C}\right)-\theta \left(\hbar \omega -\Delta_{C}\right)\right]\\ & & -\sigma_{0}\frac{v_{0/T}^{2}}{\left|c_{D}\right|\hbar \omega }\left[\theta \left(\hbar \omega -\Delta +E_{P,D}\right)-\theta \left(\hbar \omega -\Delta_{D}\right)\right]. \end{array}$$

The plot of $\sigma_{xy,\mathrm{pump}}\left(\omega \right)$ for MoS_2_, with a pump-driven photo-induced charge density $n_{\mathrm{pump}}=0.002/V_{\mathrm{cell}}$ ($\bar{p}a\approx 0.171$), is shown in Figure 3b, showing how a valley-selective population due to a circularly polarized pumping gives rise to a finite off-diagonal component (and hence to a finite Faraday/Kerr effect), not only at the A-exciton energy, $\Delta_{A}$, but also at the C-exciton energy, $\Delta_{C}$, and at another energy range, $\Delta_{D}$, corresponding to $\Delta_{D}\approx \Delta_{C}-\Delta_{A}$, net of the exciton binding energy. We predict thus an opposite sign of the off-diagonal component of the optical tensor (and hence an opposite Faraday/Kerr rotation) at the energies $\Delta_{C}$, $\Delta_{D}$ compared with the one predicted at the A-exciton energy scale. The absolute intensity of these additional features in the off-diagonal component of the optical tensor is found:

(52) $$\begin{array}{ccc} I_{C} & = & \sigma_{0}\frac{v_{T/B}^{2}}{\hbar c_{C}\Delta_{C}}E_{P,C}=4\pi a^{2}\sigma_{0}\frac{v_{T/B}^{2}}{\hbar \Delta_{C}}n_{\mathrm{pump}}, \end{array}$$

(53) $$\begin{array}{ccc} I_{D} & = & \sigma_{0}\frac{v_{0/T}^{2}}{\hbar c_{D}\Delta_{D}}E_{P,D}=4\pi a^{2}\sigma_{0}\frac{v_{0/T}^{2}}{\hbar \Delta_{D}}n_{\mathrm{pump}}. \end{array}$$

The expression of Equations (52) and (53) is formally identical at Equation (47) for $I_{A}$, upon changing the proper variables, with the noticeable difference of a factor 2. This is due to the fact that the strength of $\sigma_{xy,\mathrm{pump}}\left(\omega \right)$ at $\omega \approx \Delta_{A}$ profits from the presence of the pump-driven charge in *both* the conduction and valence bands. On the other hand, the onset of a finite off-diagonal component, $\sigma_{xy,\mathrm{pump}}\left(\omega \right)$, at the energies $\omega \approx \Delta_{C}$, $\omega \approx \Delta_{D}$ is related in an independent way only to the pump-driven charge in the conduction band and in the valence band, respectively. This complex multi-peak structure of the pump-induced Faraday/Kerr effect opens an interesting perspective, not only for characterizing and proving the onset of these effects, but also for harvesting them for multi-frequency operative devices.

## 5. Time-Dependence

In the previous section, we have shown, using an appropriate three-band model, how a valley-selective population driven by a circularly polarized pump can give rise to an off-diagonal component of the optical tensor, and hence to a finite Faraday/Kerr optical rotation at *three* characteristic energies, related to the A-exciton, the C-exciton, and to another energy scale governed by the optical transitions between the lowest conduction band and high-energy conduction band, roughly determined by the energy difference between the A and C-exciton.

Most notable, in this description, is the absence of any Faraday/Kerr signature at the B-exciton energy. This is essentially due to the strong light-polarization/orbital/valley/spin entanglement, so that a circularly polarized pumping tuned at the A-exciton resonance induces a valley/spin selective population. More, in particular, left-circularly polarized light tuned at the A-exciton resonance, in the absence of scattering, triggers particle–hole transitions uniquely at the K valley and uniquely for spin-up electrons, making thus an optical unbalance only in the spin-up sector. This scenario gives rise to a finite Faraday/Kerr signature only close to the energies $\omega \approx \Delta_{A}$, $\omega \approx \Delta_{C}$, and $\omega \approx \Delta_{D}$. Such a snapshot is valid, however, only on a short time scale, before impurity and many-body scattering can cause spin-flip and/or intervalley processes.

In order to gain an insight into how many-body scattering can affect the magneto-optical Faraday/Kerr properties induced by circularly polarized pumping, we consider the charge density in each relevant band that is involved in the time-dynamics. We denote thus $n_{3z^{2}-r^{2},\sigma }\left(\nu \right)$ as the charge density in the lowest-energy conduction band with $d_{3z^{2}-r^{2}}$-orbital and σ-spin character at the ν valley, $n_{R,↑}\left(K\right)$ as the charge (hole) density in the valence band at the K valley with spin-up, and $n_{L,↓}\left(K^{\prime }\right)$ as the charge (hole) density in the valence band at the K′ valley with spin-down. Neglecting the frequency-resolved details of each optical feature, we can estimate the “Faraday/Kerr” intensity of each spectral feature as:

(54) $$\begin{array}{ccc} I_{A}\left(t\right) & \approx & n_{3z^{2}-r^{2},↑}\left(K,t\right)+n_{R,↑}\left(K,t\right)-n_{3z^{2}-r^{2},↓}\left(K^{\prime },t\right)-n_{L,↓}\left(K^{\prime },t\right), \end{array}$$

(55) $$\begin{array}{ccc} I_{C}\left(t\right) & \approx & n_{R,↑}\left(K,t\right)-n_{L,↓}\left(K^{\prime },t\right), \end{array}$$

(56) $$\begin{array}{ccc} I_{D}\left(t\right) & \approx & n_{3z^{2}-r^{2},↑}\left(K,t\right)-n_{3z^{2}-r^{2},↓}\left(K^{\prime },t\right), \end{array}$$

(57) $$\begin{array}{ccc} I_{B}\left(t\right) & \approx & n_{3z^{2}-r^{2},↓}\left(K,t\right)-n_{3z^{2}-r^{2},↑}\left(K^{\prime },t\right). \end{array}$$

Here, following the analysis for the other features, we have properly estimated the intensity of a spectral feature at the energy corresponding to the B-exciton as resulting by the $d_{R,↓}\left(K\right)↔d_{3z^{2}-r^{2},↓}\left(K\right)$ and $d_{L,↑}\left(K^{\prime }\right)↔d_{3z^{2}-r^{2},↑}\left(K^{\prime }\right)$, and hence governed by the time-dynamics of $n_{3z^{2}-r^{2},↓}\left(K\right)$ and $n_{3z^{2}-r^{2},↑}\left(K^{\prime }\right)$. In all the cases, we have taken into account that, due to the orbital/spin/valley entanglement, similar processes at K′ cancel the contributions at the K valley. Assuming an initial pumping with left-circularly polarized photons resonant at the A-exciton energy, we model at t=0 the respective charge density as $n_{3z^{2}-r^{2},↑}\left(K,0\right)=n_{R,↑}\left(K,0\right)=n_{\mathrm{pump}}$, $n_{3z^{2}-r^{2},↓}\left(K,0\right)=n_{3z^{2}-r^{2},↓}\left(K^{\prime },0\right)=n_{3z^{2}-r^{2},↑}\left(K^{\prime },0\right)=n_{L,↓}\left(K^{\prime },0\right)=0$. These conditions reproduce the static results discussed in the previous section.

**Figure 4:**
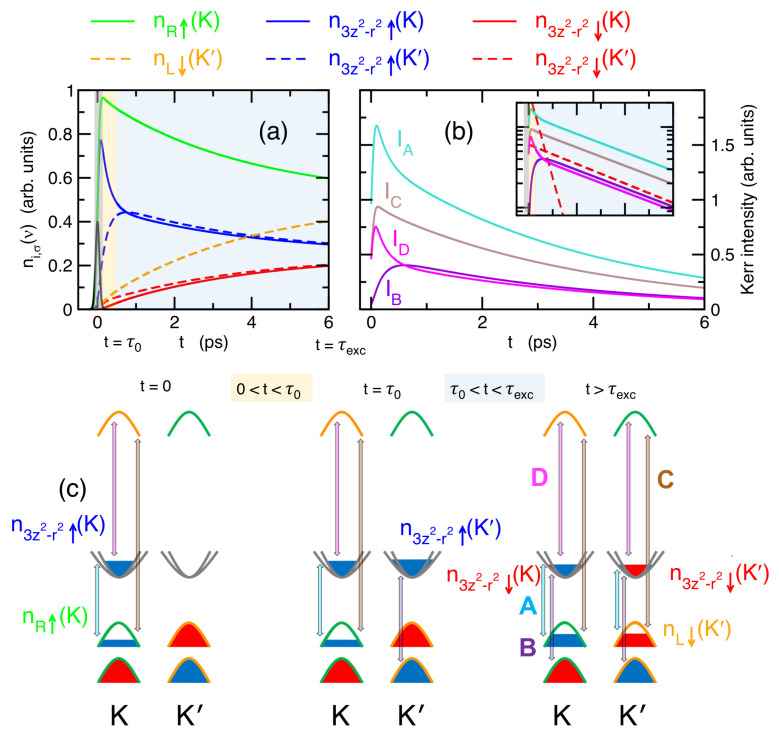
(**a**) Time-dependence of the pump-driven charge densities, $n_{i,\sigma }\left(\nu \right)$, upon a Gaussian pump with width ∼50 fs (black solid line). Legend for different charge densities is reported above. (**b**) Corresponding time-dependence of the integrated Faraday/Kerr intensities of the different spectral features associated with different optical interband transitions. Inset: the same on a linear-log plot, where the red dashed lines represent the exponential behaviors’ decay rate, $2\tau_{0}$ and $2\tau_{\mathrm{exc}}$, respectively. (**c**) Sketch of the spin-resolved charge densities, $n_{i,\sigma }\left(\nu \right)$, in the conduction and valence bands at the K and K′ points, along with the main optical transitions in the different time regimes, as outlined in the panels above.

Recombination processes, related to annihilation of particle–hole excitations, are known to occur on a very long time scale. On the other hand, the off-diagonal term, $\sigma_{xy,\mathrm{pump}}\left(\omega \right)$, of the optical tensor is expected to vanish in a much shorter time scale when scattering processes redistribute the charge in both the conduction and valence bands, giving identical populations in the K, K′ valley. Neglecting the very weak intravalley spin-flip processes, two main scattering channels have been identified in this scenario [16,34,35,36,41,59,60]: (*i*) an interband spin-conserving scattering, mediated by electron–phonon coupling and/or impurities, where the charge-density of the conduction band with given spin is scattered for a valley, ν, to the opposite valley, −ν. This process is hampered in the valence band due to the spin-splitting [34,36,41]; and (ii) spin-flip intervalley exchange, where a particle–hole couple in a given valley with given spin is scattered into the opposite valley with reverse spin [36,60,61]. According to this picture, we model in a compact way the time dynamics of the pump-driven charges with a set of coupled equations:

(58) $$\begin{array}{ccc} \frac{dn_{3z^{2}-r^{2},↑}\left(K\right)}{dt} & = & \alpha f\left(t\right)-\frac{min\left[n_{3z^{2}-r^{2},↑}\left(K\right),n_{R,↑}\left(K\right)\right]-min\left[n_{3z^{2}-r^{2},↓}\left(K^{\prime }\right),n_{L,↓}\left(K^{\prime }\right)\right]}{\tau_{\mathrm{exc}}}\\ & & -\frac{n_{3z^{2}-r^{2},↑}\left(K\right)-n_{3z^{2}-r^{2},↑}\left(K^{\prime }\right)}{\tau_{0}}, \end{array}$$

(59) $$\begin{array}{ccc} \frac{dn_{3z^{2}-r^{2},↓}\left(K\right)}{dt} & = & -\frac{n_{3z^{2}-r^{2},↓}\left(K\right)-n_{3z^{2}-r^{2},↓}\left(K^{\prime }\right)}{\tau_{0}}, \end{array}$$

(60) $$\begin{array}{ccc} \frac{dn_{R,↑}\left(K\right)}{dt} & = & \alpha f\left(t\right)-\frac{min\left[n_{3z^{2}-r^{2},↑}\left(K\right),n_{R,↑}\left(K\right)\right]-min\left[n_{3z^{2}-r^{2},↓}\left(K^{\prime }\right),n_{L,↓}\left(K^{\prime }\right)\right]}{\tau_{\mathrm{exc}}}, \end{array}$$

(61) $$\begin{array}{ccc} \frac{dn_{3z^{2}-r^{2},↑}\left(K^{\prime }\right)}{dt} & = & -\frac{n_{3z^{2}-r^{2},↑}\left(K^{\prime }\right)-n_{3z^{2}-r^{2},↑}\left(K\right)}{\tau_{0}}, \end{array}$$

(62) $$\begin{array}{ccc} \frac{dn_{3z^{2}-r^{2},↓}\left(K^{\prime }\right)}{dt} & = & -\frac{min\left[n_{3z^{2}-r^{2},↓}\left(K^{\prime }\right),n_{L,↓}\left(K^{\prime }\right)\right]-min\left[n_{3z^{2}-r^{2},↑}\left(K\right),n_{R,↑}\left(K\right)\right]}{\tau_{\mathrm{exc}}}\\ & & -\frac{n_{3z^{2}-r^{2},↓}\left(K^{\prime }\right)-n_{3z^{2}-r^{2},↓}\left(K\right)}{\tau_{0}}, \end{array}$$

(63) $$\begin{array}{ccc} \frac{dn_{L,↓}\left(K^{\prime }\right)}{dt} & = & -\frac{min\left[n_{3z^{2}-r^{2},↓}\left(K^{\prime }\right),n_{L,↓}\left(K^{\prime }\right)\right]-min\left[n_{3z^{2}-r^{2},↑}\left(K\right),n_{R,↑}\left(K\right)\right]}{\tau_{\mathrm{exc}}}, \end{array}$$

where f(t) is the profile of the pump pulse, α is related to the absorption coefficient, and $\tau_{\mathrm{exc}}$, $\tau_{0}$ are the scattering rates of the two processes discussed above. The factors $min[n_{3z^{2}-r^{2},↑}\left(K\right),n_{R,↑}\left(K\right)]$ and $min[n_{3z^{2}-r^{2},↓}\left(K^{\prime }\right),n_{L,↓}\left(K^{\prime }\right)]$ take into account that the intervalley exchange scattering requires the presence of both particle–hole changes in the conduction and valence bands. We take in the representative values $\tau_{0}=200$ fs [35,59] and $\tau_{\mathrm{exc}}=1.8$ ps [20,61]. The time dynamics of the different charge densities, $n_{i,\sigma }\left(\nu \right)$, is shown in Figure 4a, and the corresponding time-dependence of the Kerr intensity of the different spectral features in Figure 4b, whereas Figure 4c depicts some representative time-snapshots of $n_{i,\sigma }\left(\nu \right)$. Notice that, in order to focus on the time dynamics, we plot here only the dependence of $I_{\mu }\left(t\right)$ on the time-dependent charge densities, $n_{i,\sigma }\left(\nu ,t\right)$, neglecting the current operator matrix elements and other time-independent factors, so that the relative ratio of the intensities here is not meant to be representative of the experimental ratio. The overall behavior of $n_{i,\sigma }\left(\nu \right)$ and $I_{A}\left(t\right)$ that we obtain is in very good agreement with the results by Ref. [36], performed with ab initio techniques.

We can identify three main regimes. (*i*) A short time scale, $t\ll \tau_{0}$ (gray areas, left panel of Figure 4c), where the charge populations are mainly determined by the driving pump, with a significant population of only $n_{3z^{2}-r^{2},↑}\left(K\right)$ and $n_{R,↑}\left(K\right)$. This is reflected in a sharp onset of the Faraday/Kerr intensities $I_{A}\left(t\right)$, $I_{C}\left(t\right)$, and $I_{D}\left(t\right)$. (ii) Soon after this scenario, in the short time range $t∼\tau_{0}$ (yellow areas, middle panel of Figure 4c), the impurity/electron–phonon scattering has a main effect of a depletion of $n_{3z^{2}-r^{2},↑}\left(K\right)$ due a redistribution of the spin-up conduction electrons towards $n_{3z^{2}-r^{2},↑}\left(K^{\prime }\right)$. As a consequence, a sharp decrease of $I_{A}\left(t\right)\propto e^{-t/2\tau_{0}}$ and $I_{D}\left(t\right)\propto e^{-t/2\tau_{0}}$ is predicted, whereas the finite valley population, $n_{3z^{2}-r^{2},↑}\left(K^{\prime }\right)$, gives rise to a finite (delayed) intensity of $I_{B}\left(t\right)$. (iii) In the time regime $\tau_{0}\ll t∼\tau_{\mathrm{exc}}$ (light blue areas, right panel of Figure 4c), the key many-body process is the exchange scattering (assisted by the impurity/electron–phonon one), leading towards a slower equal spin population of each conduction and valence band. The total spectral intensities in this regime scale as $I_{\mu }\left(t\right)\propto e^{-t/2\tau_{\mathrm{exc}}}$. The final steady state (right panel of Figure 4c) is, however, reached only for $t\gg \tau_{\mathrm{exc}}$. In this regime, the contributions of off-diagonal elements of the optical tensor at K and K′ cancel out, and any Faraday/Kerr effect vanishes. Note that the transient Faraday/Kerr intensity at the B-exciton energy range is a by-product of the finite valley-K′ population of $n_{3z^{2}-r^{2},↑}$. In a similar way as this valley-transient population is expect to give rise to a Faraday/Kerr effect at the energy $\Delta_{B}$ associated with optical transitions between $n_{L,↑}\left(K^{\prime }\right)$ and $n_{3z^{2}-r^{2},↑}\left(K^{\prime }\right)$, we can expect the appearance of further Faraday/Kerr features at the energies associated with optical transitions between $n_{L,↑}\left(K^{\prime }\right)$ and $n_{R,↑}\left(K^{\prime }\right)$, and between $n_{3z^{2}-r^{2},↑}\left(K^{\prime }\right)$ and $n_{R,↑}\left(K^{\prime }\right)$. We denote these transitions as $\Delta_{C}^{\prime }$ and $\Delta_{D}^{\prime }$, whose Faraday/Kerr spectral intensity scales, assuming pumping tuned at the A-exciton resonance, are $I_{C}^{\prime }\left(t\right)\propto I_{C}\left(t\right)$ and $I_{D}^{\prime }\left(t\right)\propto I_{D}\left(t\right)$. The band-parameters determining the detailed spectral properties of these features are also listed in Table A1.

## 6. Conclusions

In summary, in the present paper we have addressed in an analytical way the onset of a finite Faraday/Kerr effect in single-layer transition-metal dichalcogenides upon pumping with circularly polarized light at the A-resonance. With this aim, we have introduced a proper three-band analytical k·p model that retains all the orbital complexity of the original band-structure and of the optical selection rules. We have shown how a pump-driven spin/valley selective population gives rise to a finite off-diagonal component of the optical tensor responsible for different spectral features in the Faraday/Kerr optical rotation. We recover the signature of a Faraday/Kerr rotation in the proximity of pump energy at the A-exciton resonance, in accordance with the available experimental and theoretical findings [2,19,20,21,22,23,39], and we predict the onset of a Faraday/Kerr signal at the C-exciton resonance and at a further energy scale related to the high-energy conduction band. These predictions can guide future experimental investigation, spanning also the different families of $MX_{2}$ TMDs. We have further modeled the effects of time-dynamics driven by many-body scattering, and the consequent emerging of additional transient Faraday/Kerr optical features. Our results provide suitable compact modeling for describing the magneto-optical properties induced in transition-metal dichalcogenides by circularly polarized pumping, in terms of few simple intuitive representative parameters.

## Data Availability

Data are contained within the article .

## Appendix A. Mapping k·p Band Parameters in Terms of TB Parameters

Below, we summarize useful analytical expressions for the band-parameters of the k·p Hamiltonian in Equations (6) and (7) in terms of the original tight-binding parameters provided in Ref. [54].

We have explicitly:

(A1) $$\begin{array}{ccc} E_{0} & = & \epsilon_{1}-3\left(t_{0}-2r_{0}+u_{0}\right), \end{array}$$

(A2) $$\begin{array}{ccc} E_{T} & = & \epsilon_{2}-\frac{3}{2}\left(t_{11}+t_{22}-4r_{11}+u_{11}+u_{22}\right)+2\sqrt{3}r_{12}+3\sqrt{3}t_{12}-3\sqrt{3}u_{12}, \end{array}$$

(A3) $$\begin{array}{ccc} E_{B} & = & \epsilon_{2}-\frac{3}{2}\left(t_{11}+t_{22}-4r_{11}+u_{11}+u_{22}\right)+2\sqrt{3}r_{12}-3\sqrt{3}t_{12}+3\sqrt{3}u_{12}, \end{array}$$

(A4) $$\begin{array}{ccc} a_{0} & = & \frac{3(t_{0}-6r_{0}+4u_{0})}{4}, \end{array}$$

(A5) $$\begin{array}{ccc} a_{T} & = & \frac{3}{8}\left(t_{11}+4u_{11}+t_{22}+4u_{22}\right)-\frac{9}{2}r_{11}-\frac{3\sqrt{3}}{2}r_{12}-\frac{3\sqrt{3}}{4}t_{12}+3\sqrt{3}u_{12}, \end{array}$$

(A6) $$\begin{array}{ccc} a_{B} & = & \frac{3}{8}\left(t_{11}+4u_{11}+t_{22}+4u_{22}\right)-\frac{9}{2}r_{11}-\frac{3\sqrt{3}}{2}r_{12}+\frac{3\sqrt{3}}{4}t_{12}-3\sqrt{3}u_{12}, \end{array}$$

(A7) $$\begin{array}{ccc} v_{0/T} & = & -\frac{3\sqrt{3}}{2\sqrt{2}}t_{2}+\frac{3\sqrt{3}}{\sqrt{2}}u_{2}+\frac{3}{2\sqrt{2}}t_{1}-\frac{3(r_{1}-r_{2})}{\sqrt{2}}+\frac{3}{\sqrt{2}}u_{1}, \end{array}$$

(A8) $$\begin{array}{ccc} v_{0/B} & = & -\frac{3\sqrt{3}}{2\sqrt{2}}t_{2}+\frac{3\sqrt{3}}{\sqrt{2}}u_{2}-\frac{3}{2\sqrt{2}}t_{1}+\frac{3(r_{1}-r_{2})}{\sqrt{2}}-\frac{3}{\sqrt{2}}u_{1}, \end{array}$$

(A9) $$\begin{array}{ccc} v_{T/B} & = & \frac{3\sqrt{3}}{4}\left(t_{11}-t_{22}-2u_{11}+2u_{22}\right). \end{array}$$

The terms ${\bar{a}}_{T,\sigma }$, ${\bar{a}}_{0,\sigma }$, and ${\bar{a}}_{B,\sigma }$ are related to the dispersion mass of each band, and they are obtained within the k·p context as:

(A10) $$\begin{array}{ccc} {\bar{a}}_{T,↑} & = & a_{T}+\gamma_{T/B,↑}+\gamma_{T/0,↑}, \end{array}$$

(A11) $$\begin{array}{ccc} {\bar{a}}_{0,↑} & = & a_{0}+\gamma_{0/B,↑}-\gamma_{T/0,↑}, \end{array}$$

(A12) $$\begin{array}{ccc} {\bar{a}}_{B,↑} & = & a_{B}-\gamma_{T/B,↑}-\gamma_{0/B,↑}, \end{array}$$

(A13) $$\begin{array}{ccc} {\bar{a}}_{T,↓} & = & a_{T}+\gamma_{T/B,↓}+\gamma_{T/0,↓}, \end{array}$$

(A14) $$\begin{array}{ccc} {\bar{a}}_{0,↓} & = & a_{0}+\gamma_{0/B,↓}-\gamma_{T/0,↓}, \end{array}$$

(A15) $$\begin{array}{ccc} {\bar{a}}_{B,↓} & = & a_{B}-\gamma_{T/B,↓}-\gamma_{0/B,↓}, \end{array}$$

and where

(A16) $$\begin{array}{ccc} \gamma_{T/B,\sigma } & = & \frac{v_{T/B}^{2}}{E_{T}-E_{B}+2\lambda I_{\sigma }}, \end{array}$$

(A17) $$\begin{array}{ccc} \gamma_{T/0,\sigma } & = & \frac{v_{0/T}^{2}}{E_{T}-E_{0}+\lambda I_{\sigma }}, \end{array}$$

(A18) $$\begin{array}{ccc} \gamma_{0/B,\sigma } & = & \frac{v_{0/B}^{2}}{E_{0}-E_{B}+\lambda I_{\sigma }}. \end{array}$$

The spin-dependent single-particle energies at the K point read thus:

(A19) $$\begin{array}{ccc} \epsilon_{T,↑} & = & E_{T}-\lambda , \end{array}$$

(A20) $$\begin{array}{ccc} \epsilon_{0,↑} & = & E_{0}, \end{array}$$

(A21) $$\begin{array}{ccc} \epsilon_{B,↑} & = & E_{B}+\lambda , \end{array}$$

(A22) $$\begin{array}{ccc} \epsilon_{T,↓} & = & E_{T}+\lambda , \end{array}$$

(A23) $$\begin{array}{ccc} \epsilon_{0,↓} & = & E_{0}, \end{array}$$

(A24) $$\begin{array}{ccc} \epsilon_{B,↓} & = & E_{B}-\lambda , \end{array}$$

and the characteristic interband optical edges:

(A25) $$\begin{array}{ccc} \Delta_{A} & = & \epsilon_{0,↑}-\epsilon_{B,↑}, \end{array}$$

(A26) $$\begin{array}{ccc} \Delta_{B} & = & \epsilon_{0,↓}-\epsilon_{B,↓}, \end{array}$$

(A27) $$\begin{array}{ccc} \Delta_{C} & = & \epsilon_{T,↑}-\epsilon_{B,↑}, \end{array}$$

(A28) $$\begin{array}{ccc} \Delta_{D} & = & \epsilon_{T,↑}-\epsilon_{0,↑}, \end{array}$$

(A29) $$\begin{array}{ccc} \Delta_{C^{\prime }} & = & \epsilon_{T,↓}-\epsilon_{B,↓}, \end{array}$$

(A30) $$\begin{array}{ccc} \Delta_{D^{\prime }} & = & \epsilon_{T,↓}-\epsilon_{0,↓}. \end{array}$$

In Table A1, we summarize the numerical values of our three-band k·p model obtained from the initial tight-binding parameters of Ref. [54]:

**Table A1:** Characteric electron and optical parameters for different families of semiconducting transition-metal dichalcogenides. All parameters are in units of eV.

	MoS_2_	MoSe_2_	MoTe_2_	WS_2_	WSe_2_	WTe_2_
λ	0.073	0.091	0.107	0.211	0.228	0.237
$E_{T}$	3.451	3.056	2.525	3.933	3.443	2.871
$E_{0}$	1.595	1.482	1.113	1.749	1.565	1.132
$E_{B}$	−0.062	0.053	0.041	−0.057	0.024	0.065
$\epsilon_{T,↑}$	3.378	2.965	2.418	3.722	3.215	2.634
$\epsilon_{0,↑}$	1.595	1.482	1.113	1.749	1.565	1.132
$\epsilon_{B,↑}$	0.011	0.144	0.148	0.154	0.252	0.302
$\epsilon_{T,↓}$	3.524	3.147	2.632	4.144	3.671	3.108
$\epsilon_{0,↓}$	1.595	1.482	1.113	1.749	1.565	1.132
$\epsilon_{B,↓}$	−0.135	−0.038	−0.066	−0.268	−0.204	−0.172
$\Delta_{A}$	1.584	1.338	0.965	1.595	1.313	0.830
$\Delta_{B}$	1.730	1.520	1.179	2.017	1.769	1.304
$\Delta_{C}$	3.367	2.821	2.270	3.569	2.963	2.332
$\Delta_{D}$	1.783	1.483	1.305	1.973	1.650	1.502
$\Delta_{C^{\prime }}$	3.659	3.185	2.698	4.413	3.875	3.280
$\Delta_{D^{\prime }}$	1.929	1.665	1.519	2.395	2.106	1.976
$a_{T}$	−1.190	−1.170	−1.142	−1.305	−1.310	−1.221
$a_{0}$	−0.493	−0.411	−0.012	−0.586	−0.455	−0.212
$a_{B}$	1.060	0.921	0.578	1.299	1.119	0.860
${\bar{a}}_{T,↑}$	−0.800	−0.774	−0.768	−0.836	−0.829	−0.816
${\bar{a}}_{0,↑}$	0.961	0.800	0.873	1.512	1.375	1.624
${\bar{a}}_{B,↑}$	−0.777	−0.675	−0.667	−1.245	−1.161	−1.346
${\bar{a}}_{T,↓}$	−0.831	−0.819	−0.826	−0.925	−0.941	−0.932
${\bar{a}}_{0,↓}$	0.838	0.654	0.710	1.071	0.902	0.953
${\bar{a}}_{B,↓}$	−0.637	−0.505	−0.470	−0.756	−0.628	−0.615
$v_{0/B}$	−1.545	−1.292	0.956	−1.850	−1.565	−1.244
$v_{0/T}$	−0.306	−0.236	0.286	−0.303	−0.246	−0.204
$v_{T/B}$	−1.065	−1.001	0.841	−1.228	−1.148	−0.938
$c_{A}$	1.739	1.475	1.540	2.757	2.535	2.969
$c_{B}$	1.474	1.160	1.180	1.828	1.530	1.568
$c_{C}$	−0.023	−0.098	−0.101	0.409	0.332	0.530
$c_{D}$	−1.762	−1.573	−1.641	−2.348	−2.204	−2.439
$c_{C^{\prime }}$	−0.194	−0.314	−0.356	−0.168	−0.313	−0.317
$c_{D^{\prime }}$	−1.669	−1.473	−1.536	−1.996	−1.843	−1.884
